# Effect of platelet-rich fibrin on postoperative healing, edema, and pain in orthodontic premolar extractions: a split-mouth randomized clinical trial

**DOI:** 10.21142/2523-2754-1404-2026-317

**Published:** 2026-10-01

**Authors:** Paul Efraín Medina Siguenza, Magdalena Molina Barahona

**Affiliations:** 1 Postgraduate Program in Periodontology and Surgical Implantology, Catholic University of Cuenca. Cuenca, Ecuador. pemedinas47@est.ucacue.edu.ec Postgraduate Program in Periodontology and Surgical Implantology Catholic University of Cuenca Cuenca Ecuador pemedinas47@est.ucacue.edu.ec; 2 Department of Maxillofacial Radiology, School of Dentistry, Catholic University of Cuenca. Cuenca, Ecuador. rocio.molina@ucacue.edu.ec Department of Maxillofacial Radiology School of Dentistry Catholic University of Cuenca Cuenca Ecuador rocio.molina@ucacue.edu.ec

**Keywords:** platelet-rich fibrin, wound healing, tooth extraction, postoperative pain, fibrina rica en plaquetas, cicatrización de heridas, extracción dental, dolor posoperatorio

## Abstract

**Objective:**

To evaluate the effect of platelet-rich fibrin (PRF) on soft tissue healing, facial edema, and postoperative pain following orthodontic premolar extractions using a split-mouth randomized design.

**Methods:**

A randomized controlled split-mouth clinical trial (ClinicalTrials.gov: NCT06900036; Ethics Committee: CEISH-UCACUE-031) was conducted in ten healthy patients (mean age: 24.6 ± 5.8 years; range: 17-38) undergoing bilateral premolar extractions, resulting in 36 extraction sockets (19 PRF, 17 control). Outcomes were assessed on days 0, 3, 7, and 14 using the Landry healing index, the Neupert method for facial edema, and the Visual Analog Scale (VAS) for pain. Linear mixed models (LMM) with Bonferroni correction were applied for continuous variables; Friedman and Wilcoxon signed-rank tests for ordinal variables. Significance was set at α = 0.05**.**

**Results:**

No statistically significant differences were found between groups in any evaluated parameter (p > 0.05). Time significantly affected most inflammatory indicators (p < 0.05), reflecting progressive edema resolution in both groups. The time × group interaction was not significant (p > 0.05). Healing reached good-to-excellent levels from day 3 in both groups. The PRF group showed a non-significant trend toward lower early pain scores.

**Conclusion:**

In low-morbidity surgical contexts, PRF did not significantly improve soft tissue healing, facial edema, or postoperative pain compared with spontaneous healing. Its favorable safety profile supports further evaluation in higher-morbidity settings; however, the present results do not support its routine use in low-complexity orthodontic extractions. Future studies with greater statistical power, higher-morbidity procedures, and three-dimensional evaluation tools are needed to better define its clinical utility.

## RESUMEN

**Objetivo:**

Evaluar el efecto de la fibrina rica en plaquetas (PRF) sobre la cicatrización de tejidos blandos, el edema facial y el dolor posoperatorio tras extracciones de premolares por indicación ortodóncica, mediante un diseño de boca dividida.

**Métodos:**

Se realizó un ensayo clínico aleatorizado controlado con diseño de boca dividida (ClinicalTrials.gov: NCT06900036; Comité de Ética: CEISH-UCACUE-031) en diez pacientes sanos (edad media: 24,6 ± 5,8 años; rango: 17-38) sometidos a extracciones bilaterales de premolares, y se obtuvo 36 alvéolos: 19 en el grupo PRF y 17 en el grupo control. Los resultados se evaluaron en los días 0, 3, 7 y 14 mediante el índice de cicatrización de Landry, el método de Neupert para el edema facial y la Escala Visual Analógica (EVA) para el dolor. Se aplicaron modelos lineales mixtos (MLM) con corrección de Bonferroni para variables continuas, así como pruebas de Friedman y Wilcoxon para variables ordinales. El nivel de significación se estableció en α = 0,05.

**Resultados:**

No se observaron diferencias estadísticamente significativas entre los grupos en ninguno de los parámetros evaluados (p > 0,05). El factor tiempo influyó significativamente en la mayoría de los indicadores inflamatorios (p < 0,05), lo que refleja una resolución progresiva del edema en ambos grupos. La interacción tiempo × grupo no fue significativa (p > 0,05). La cicatrización alcanzó niveles de buena a excelente a partir del día 3 en ambos grupos. El grupo PRF mostró una tendencia no significativa hacia puntuaciones de dolor más bajas durante la fase posoperatoria temprana.

**Conclusión:**

En contextos quirúrgicos de baja morbilidad, la PRF no mejoró significativamente la cicatrización de tejidos blandos, el edema facial ni el dolor posoperatorio en comparación con la cicatrización espontánea. Su perfil de seguridad favorable justifica su evaluación en contextos de mayor morbilidad; sin embargo, los presentes resultados no respaldan su uso rutinario en extracciones ortodóncicas de baja complejidad.

## INTRODUCTION

Tooth extractions are one of the most common surgical procedures in dental practice and trigger a repair process involving soft tissues and the alveolar bone. Following tooth extraction, physiological remodeling of the alveolus occurs rapidly during the first few months, a period in which a significant reduction in the alveolar ridge may occur both horizontally and vertically. ^1^^,^^2^ This tissue repair process is accompanied by local changes that influence the clinical outcome of the surgical site. ^3^

The immediate postoperative period is characterized by an acute inflammatory response that includes pain, facial edema, and, in some cases, trismus, factors that directly impact the patient’s comfort and quality of life. ^4^^-^^7^ The intensity of these manifestations depends on the degree of surgical trauma and the individual biological response, which has prompted the search for therapeutic strategies aimed at optimizing healing and reducing postoperative morbidity.

In this context, autologous platelet concentrates have been proposed as biological alternatives with regenerative potential. Platelet-rich fibrin (PRF), first described by Choukroun et al. ^8^, is obtained without anticoagulants or activators, preserving its autologous nature and three-dimensional fibrin architecture. ^9^^,^^10^ This matrix acts as a scaffold for cell migration and as a reservoir for the progressive release of bioactive factors.

PRF contains a high concentration of platelets and leukocytes capable of releasing growth factors such as PDGF, TGF-β1, VEGF, IGF, and EGF, which are involved in cell proliferation, angiogenesis, and extracellular matrix synthesis. ^11^^-^^13^ Likewise, leukocytes contribute to the modulation of the inflammatory response by secreting interleukins such as IL-4 and IL-10, promoting a biological environment conducive to tissue regeneration. ^14^^,^^15^ These properties support its use as an adjunct in post-extraction healing.

The available clinical evidence, based on randomized controlled trials and systematic reviews, has shown that PRF can improve soft tissue healing, ^16^^-^^19^ decrease pain perception, ^16^^,^^20^ and reduce postoperative edema, ^7^^,^^21^ as well as the incidence of healing-related complications. ^16^^,^^17^^,^^19^^,^^22^ Complementarily, Arora et al. ^23^ reported benefits in the clinical course of post-extraction sites. Proposed mechanisms include the protection of nerve endings, associated with reduced pain perception in early phases, as well as its immunomodulatory effect, which limits fluid extravasation and contributes to the reduction of edema. Furthermore, its function as a physical barrier promotes clot stabilization and earlier epithelialization of the socket. ^24^^-^^26^

However, much of the available evidence comes from clinical contexts involving a higher degree of surgical trauma. In contrast, there is limited information from more controlled and standardized settings, such as orthodontic premolar extractions in young, healthy patients. This model allows for the application of split-mouth designs, reducing interindividual variability and strengthening the internal validity of the studies, ^27^ but has been scarcely explored in recent literature.

In this context, the present study was designed as a randomized clinical trial with a split-mouth design, in accordance with the CONSORT guidelines and the Declaration of Helsinki, with the aim of evaluating the effect of platelet-rich fibrin (PRF) on soft tissue healing, facial edema, and postoperative pain in premolar extractions for orthodontic indications. Secondary objectives included comparing the quality of healing between groups, analyzing the evolution of edema on days 3, 7, and 14, and evaluating differences in pain perception.

## MATERIALS AND METHODS

### Study Design

A randomized controlled clinical trial with a split-mouth design was conducted, in which each patient served as their own control through the random assignment of extraction sites to the experimental (PRF) and control groups.

The study was conducted in the Department of Dentistry at the Catholic University of Cuenca (Ecuador), following the guidelines of the CONSORT statement. The protocol was approved by the Research Ethics Committee (CEISH-UCACUE-031) and prospectively registered on ClinicalTrials.gov (NCT06900036). Participant recruitment took place between January and May 2024.

### Participants

*Inclusion criteria:*

- Healthy patients of both sexes
- Aged between 17 and 38 years
- Requirement for bilateral extraction of upper or lower premolars for orthodontic reasons
- Willingness to attend follow-up visits on days 0, 3, 7, and 14 post-surgery
- Signature of informed consent

*Exclusion criteria:*

- Presence of systemic diseases
- Immunosuppression of any etiology
- Smoking
- Pregnancy or breastfeeding
- Platelet count less than 200,000/mm³
- Recent use of medications that could interfere with the healing process

All participants signed a written informed consent form, previously approved by the institutional ethics committee.

### Sample Size Calculation and Randomization

The sample size was determined using G*Power v3.1.9.4 software (Heinrich-Heine-Universität Düsseldorf, Germany), considering a repeated-measures design in accordance with the split-mouth protocol, with an effect size of 0.25, a significance level (α) of 0.05, and a statistical power of 95%. As a result, a minimum sample size of 36 alveoli was established, assigned to the experimental (PRF) and control groups.

The random assignment of extraction sites was performed using a simple random sequence generated with Epidat software version 4.2 (Directorate General of Public Health, Regional Government of Galicia, Spain), without blocking or stratification. The sequence was generated by an independent biostatistician and applied by a clinician other than the principal investigator, randomly assigning the contralateral alveoli of each patient to the study groups.

Of the ten participants enrolled, eight underwent bilateral extraction of four premolars (two maxillary and two mandibular), while the remaining two underwent bilateral extraction of two premolars, resulting in a total of 36 extraction sockets. In patients with four extraction sites (n = 8), randomization was performed at the arch level: one full arch (maxillary or mandibular) was randomly assigned to the PRF group and the contralateral arch to the control group, such that both sockets within the assigned arch received the same intervention. In patients with two extraction sites (n = 2), contralateral sockets were individually randomized to each group. This approach yielded 19 sockets in the PRF group and 17 in the control group, an asymmetry arising from the arch-level allocation strategy rather than from participant dropout or exclusion. 

To reduce selection bias, assignment was performed prior to the surgical procedure. Patients were unaware of the treatment assignment, so the study was considered a single-blind trial. Due to the nature of the intervention, neither the surgical operator nor the clinical evaluator could be blinded, which is recognized as a potential limitation due to the risk of detection bias.

The use of a split-mouth design allowed for a reduction in interindividual variability and strengthened the internal validity of the study by comparing contralateral sites within the same patient.

The process of participant selection, allocation, follow-up, and analysis is presented in the flow diagram in accordance with the CONSORT guidelines (Figure 1).

**Figure 1:**
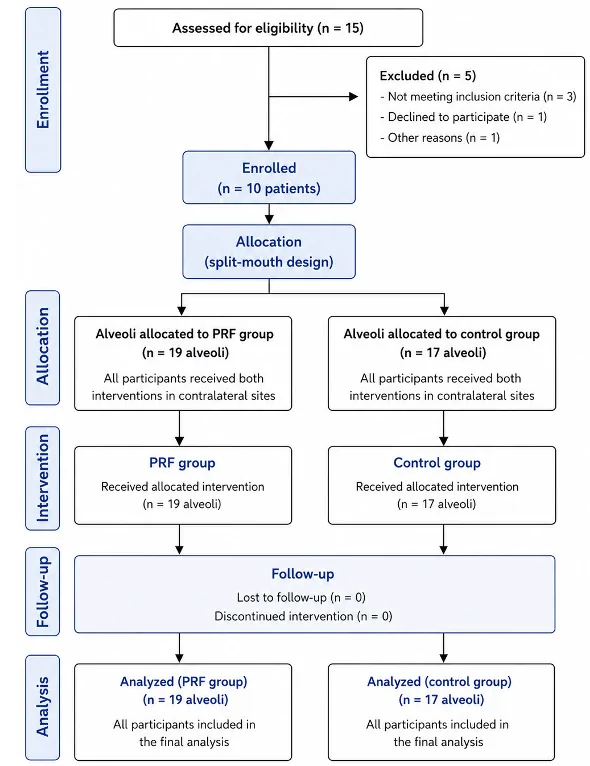
Flowchart of participant selection, allocation, intervention, follow-up, and analysis according to CONSORT guidelines. Of 10 enrolled participants, all completed the study protocol with no losses to follow-up.

### PRF Preparation

The procedure was performed using a conventional PRF preparation protocol derived from Choukroun's method. ^(8^^,^^28^ Autologous platelet-rich fibrin (PRF) was obtained via venipuncture in the antecubital vein, collecting 10 mL of blood in two sterile, additive-free glass tubes (BD Vacutainer® Push Button Blood Collection Set, 23G × –″, 12″ tube with Luer adapter; REF: 367342, Becton Dickinson, Franklin Lakes, NJ, USA). The samples were centrifuged in a FibrinFUGE 25 centrifuge (Montserrat®, Montevideo, Uruguay) at 2700 rpm for 12 minutes, without any chemical manipulation (Figure 2a). They were allowed to stand for 5 minutes. The clots were carefully isolated between the erythrocyte layer and the acellular plasma (Figure 2b). One of the clots was inserted into the alveolus as a biological matrix, while the second was gently compressed with a surgical metal box (Zimmer Biomet Dental, Palm Beach Gardens, FL, USA) to form a membrane. Both were stabilized with 3-0 catgut absorbable suture (Sutures India Pvt Ltd., Tamil Nadu, India).

**Figure 2:**
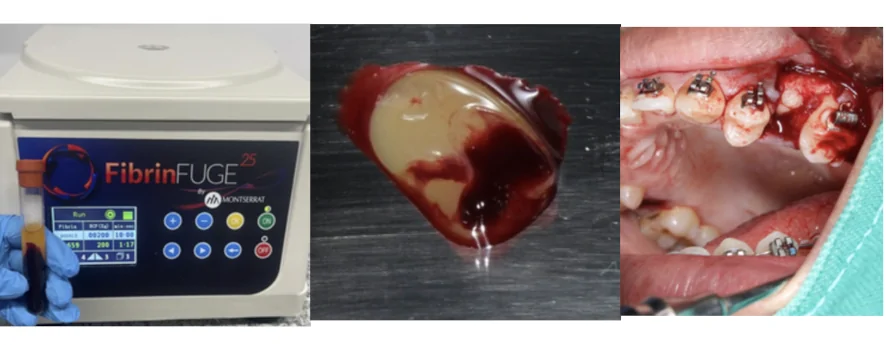
A) Blood sample after centrifugation with the FibrinFUGE 25 (Montserrat®, Montevideo, Uruguay), showing the separation of the leukocyte- and platelet-rich fibrin clot (PRF). The clot is visible as the yellowish intermediate layer situated between the platelet-poor plasma at the top and the red blood cell fraction at the bottom. This stage precedes the preparation of the membrane. B) Freshly obtained PRF clot prior to compression. The fibrin matrix appears well-formed, with visible incorporation of red blood cells at the base. This intermediate step is essential prior to the formation of a membrane suitable for insertion into the extraction socket. C) Atraumatic post-extraction socket of the upper left first premolar (tooth 2.4), following its surgical extraction for orthodontic purposes. The socket has intact cortical walls and minimal soft tissue trauma, making it a favorable site for PRF membrane placement.

### Surgical Protocol

The surgical procedures for the experimental and control groups were performed during the same operative session, ensuring comparable clinical conditions. All extractions were performed by a single experienced operator using atraumatic techniques under sterile conditions.

Local anesthesia was administered with 4% articaine with epinephrine 1:100,000 (Inibsa Dental, Barcelona, Spain). Subsequently, an intrasulcular incision was made with a No. 15 scalpel (Bard-Parker®, Aspen Surgical Products, USA), and the premolars were extracted according to orthodontic guidelines, taking care to preserve the integrity of the alveolar walls.

No adjunctive biomaterial was applied in the control sites, allowing for spontaneous healing. In the experimental sites, a PRF clot was placed inside the alveolus and a membrane derived from it was placed over the alveolar surface, acting as a biological barrier to promote healing (Figure 2c).

The site was closed using simple interrupted sutures with absorbable suture material. The only difference between the two groups was the application of PRF, with all other surgical conditions remaining constant.

The surgical procedures and the collection of clinical data were performed by a multidisciplinary team, following a standardized protocol.

### Clinical Evaluation

Clinical variables were assessed on days 0 (immediately following extraction), 3, 7, and 14 postoperatively.

Healing: This was evaluated using the Landry, Turnbull, and Howley clinical index, ^29^ which classifies the quality of healing on a scale from 1 (very poor) to 5 (excellent), considering parameters such as tissue color, bleeding, granulation tissue, epithelialization, and suppuration. The evaluation was performed by direct clinical inspection under standard lighting.

Facial edema: This was measured using the Neupert method, ^4^ by recording five facial distances from the mandibular angle to anatomical landmarks (the tragus, the lateral canthus, the nasal wing, the corner of the mouth, and the pogonion), using suture thread and a millimeter ruler. Changes were expressed as a percentage of the baseline value.

Pain: Pain was assessed using the Visual Analog Scale (VAS), ^30^ using a 10-cm horizontal line, where 0 corresponds to “no pain” and 10 to “maximum imaginable pain.” The scale was explained to each patient beforehand and completed self-reported immediately after the procedure (day 0) and at follow-up visits on days 3, 7, and 14.

### Calibration of the evaluators

All clinical records were completed by a single investigator previously trained in the application of the scales used. To ensure consistency in measurements, an intra-observer calibration process was conducted via a pilot study prior to the start of the main study.

Intra-observer agreement was assessed by repeating the measurements in a subset of cases, yielding intraclass correlation coefficients (ICC) greater than 0.80 for the continuous measurements of the Neupert method (range: 0.81-0.94) and weighted Kappa values greater than 0.75 for the Landry index, indicating good to excellent agreement according to the criteria of Landis and Koch (1977).

### Statistical Analysis

Data processing and analysis were performed using JASP software (version 0.19.0; JASP Team, 2024). The continuous variables facial distances L1 to L5 and global edema were described using the mean and standard deviation (SD); the ordinal variables healing and pain were summarized using the median and interquartile range (IQR). The denominator degrees of freedom for between-subjects effects were estimated using Satterthwaite’s method, which may yield non-integer values or values greater than the number of participants in unbalanced designs.

Given the nature of the split-mouth design, in which each participant served simultaneously as both case and control in contralateral quadrants, mixed linear models (MLM) were used to analyze the continuous variables. The model included as fixed effects time (immediate, day 3, day 7, and day 14), group (PRF vs. control), and their time × group interaction; the participant was included as a random effect to adequately model intra-individual correlation. Because multiple sockets were nested within the same participant, this approach accounts for intra-individual dependency and avoids inflation of the effective sample size, consistent with current methodological recommendations for split-mouth designs involving multiple sites per patient. The covariance structure was selected using information criteria (AIC/BIC). In the presence of significant effects, pairwise comparisons with Bonferroni correction were performed.

For ordinal variables, the Friedman test was applied to assess within-subject changes over time within each group, and the Wilcoxon signed-rank test was used for paired comparisons between groups at each time point, thereby accounting for the dependency of observations from the same patient.

## RESULTS

### Sample Characteristics

Ten patients who met the eligibility criteria were included, contributing a total of 36 alveoli, distributed between the PRF group (n = 19) and the control group (n = 17). No losses to follow-up or exclusions from the analysis were recorded.

The demographic characteristics of the sample are presented in Table 1.

**Table 1:** Baseline demographic and clinical characteristics of the participants included in the study. Data are presented as mean ± standard deviation (SD) or as absolute and relative frequencies (%)

Variable	Value
Number of patients (n)	10
Age (years), mean ± SD	24.6 ± 5.8
Age range (years)	17-38
Sex, n (%)	
- Female	6 (60%)
- Male	4 (40%)
Type of extraction, n (%)	
- Upper premolars	18 (50%)
- Lower premolars	18 (50%)
Total number of alveoli	36
PRF group, n (%)	19 (52.8%)
Control group, n (%)	17 (47.2%)

### Descriptive analysis of inflammation

In the immediate postoperative period (day 0), indicators L1, L4, and L5, as well as overall edema, exhibited wide interquartile ranges, indicating high interindividual variability in the initial inflammatory response. In contrast, L2 and L3 showed less dispersion, with values close to baseline from the first readings.

Initial inflammatory changes ranged from 0% to 9.8% depending on the indicator considered. Global edema values immediately post-surgery were 5.3% in the PRF group and 5.4% in the control group, with no clinically relevant differences.

At 72 hours, a generalized reduction in inflammation was observed, with values below 2% for most indicators. This downward trend persisted at 7 and 14 days, establishing a pattern of progressive and consistent resolution in both groups.

The L4 indicator showed the highest initial inflammatory values, followed by L1. In contrast, L2 and L3 exhibited less variability and smaller changes throughout the follow-up period.

Regarding temporal evolution, the mean values for L1 decreased from 8.2% (PRF) and 8.1% (control) in the immediate period to negative values at 14 days, indicating complete resolution of the inflammatory process relative to baseline. In L4, the initial values of 13.0% (PRF) and 15.1% (control) followed a similar trajectory, with values below baseline at the end of follow-up.

Overall edema showed a sustained reduction in both groups, from 5.3% (PRF) and 5.4% (control) in the immediate period to -7.1% and -6.1%, respectively, at 14 days.

Overall, the variability observed in the initial measurements progressively decreased, with greater variability in the early phase followed by stabilization in later stages. Complete descriptive statistics are presented in Table 2.

**Table 2:** Descriptive statistics of the percentage changes in facial edema by intervention group and time of evaluation

		Immediate		3 days		7 days		14 days	
Variable	Statistical	PRF	Control	PRF	Control	PRF	Control	PRF	Control
L1	Mean	8.2	8.1	0.4	-0.2	-5.6	-5	-15.3	-14.7
	Median	3.1	4	0	0	-10.1	-1.6	-11.7	-6.3
	Q1	-3.6	-3.8	-3.9	-13.4	-13.8	-0.1	-28.6	-4.1
	Q3	11.6	25.8	9.1	9.1	0	2.1	-3.6	1.1
	SD	15.4	19.1	8.2	16.2	10.2	2.1	15.7	6.5
L2	Mean	-1.5	-2	-0.8	0.1	-4	-5.7	-4.7	-16.4
	Median	0	0	2	0	1	0	-3	-4.8
	Q1	-4.6	-10.3	-6.7	-5.5	-12.4	1.3	-19.1	-1.4
	Q3	1.1	4	4.6	2	2.3	-1.2	9.8	0
	SD	7.6	9.5	9.5	7.8	9.6	2.3	12.3	4.3
L3	Mean	7.2	7.3	0.3	1.8	-0.8	4	-2.7	0
	Median	5.9	4.7	0	0	0	3.1	-0.9	-2
	Q1	1.9	-1	-5.4	-4.5	-10.9	7.3	-13	3.7
	Q3	6.5	9	7.8	3.6	6.7	1	4.4	2
	SD	8.7	20.1	6.6	20.6	9	5.5	9	0.04
L4	Mean	13	15.1	-0.1	2.6	2.3	-15.7	-5.9	-34.7
	Median	7.5	9.8	-1.4	1.2	1.3	-4	-3.8	-10.3
	Q1	-4	4.8	-6.7	0	1.3	-4.9	-16	-13.9
	Q3	28.2	23.3	6.9	6.5	6.7	-11.1	0	-5.4
	SD	17.4	16.2	9.7	4.8	7.7	10	10.5	17.2
L5	Mean	-0.2	-1.4	-2.3	-1.5	-1.2	-17.2	-6.7	-23
	Median	4.5	0	-5.6	-2.2	1.1	-7.7	-6.7	-11.9
	Q1	-8.9	-4.3	-7.6	-8.2	-9	-15.1	-16	-14.2
	Q3	6.5	4.5	1.1	3.3	2.3	-21.6	-2.3	-20.1
	SD	9.4	7.8	8.3	13	8.8	11.9	10.4	10.9
Edema	Mean	5.3	5.4	-0.5	0.5	-1.9	-1.3	-7.1	-6.1
	Median	6	5.7	1	1.3	2.7	-1.8	-3.3	-4.5
	Q1	2.4	2.4	0	-4.6	-9.3	-6.9	-16.1	-18.3
	Q3	8.2	8.4	1.3	2.7	3.3	5.3	0.9	3.9
	SD	7.1	5.6	5.6	5.3	7.5	9.7	10.7	13.1

Note. L1 = distance from the mandibular angle to the superior pole of the tragus; L2 = distance from the mandibular angle to the outer canthus of the eye; L3 = distance from the mandibular angle to the outer crease of the nasal wing; L4 = distance from the mandibular angle to the corner of the mouth; L5 = distance from the mandibular angle to the pogonion; Edema = overall mean of the percentage changes in the five facial measurements; SD = standard deviation; Q1 = first quartile; Q3 = third quartile. Negative values indicate a reduction in facial measurements relative to baseline, reflecting resolution of edema below pre-surgical levels.

### Inferential analysis

The mixed linear models showed no significant time × group interaction for any inflammatory indicator evaluated (L1-L5 and overall edema), indicating that the temporal pattern of inflammatory resolution was comparable between the PRF and control groups. Descriptive between-group differences were small and did not suggest clinically relevant superiority of PRF.

The time factor, however, had a significant effect on most indicators (p < 0.05), reflecting a progressive and sustained reduction in inflammation in both groups (Table 3). 

**Table 3:** Linear mixed model analysis for inflammatory markers: fixed effects of time and time × group interaction

Variable	Effect	p
L1	Time	0.001*
	Time × Group	0.96
	Residual	-
L2	Time	0.096
	Time × Group	0.682
	Residual	-
L3	Time	0.006*
	Time × Group	0.637
	Residual	-
L4	Time	<0.001*
	Time × Group	0.731
	Residual	-
L5	Time	0.038*
	Time × Group	0.87
	Residual	-
Edema	Time	0.001*
	Time × Group	0.915
	Residual	-

* p < 0.05.

### Post hoc comparisons

Since the group effect was not significant, the post hoc analysis focused on characterizing temporal differences within the combined sample. For the inflammation markers L1 and L4, significant differences were identified between the immediate period and days 3, 7, and 14 (pholm < 0.05), with positive mean differences (T1 − T2) ranging from 8.07 to 23.14 percentage points, reflecting a progressive reduction in inflammation markers throughout the follow-up period.

For L2, no temporal comparison reached statistical significance (p > 0.05). For L3, the only significant difference was observed between the immediate period and day 14 (p < 0.05), with a reduction in values over time. For L5, differences were concentrated on day 14 compared to days 3 and 7.

Overall edema showed significant differences between most of the evaluated intervals, with the exception of the comparison between days 3 and 7.

Although the general trend showed a progressive decrease in inflammation markers, isolated variations with transient increases in some specific markers were observed (negative values in T1 − T2), reflecting individual biological variability in the postoperative inflammatory response.

Detailed results are presented in Table 4.

**Table 4:** Post hoc comparisons of mean differences between assessment periods

Variable	T1	T2	Mean difference	pholm
L1	Immediate	3 days	8.067	0.014
		7 days	13.494	0.024
		14 days	23.144	0.004
	3 days	7 days	5.427	0.184
		14 days	15.077	0.024
	7 days	14 days	9.65	<0.001
L2	Immediate	3 days	-1.376	1
		7 days	1.048	1
		14 days	3.756	0.475
	3 days	7 days	2.425	0.209
		14 days	5.132	0.015
	7 days	14 days	2.707	0.475
L3	Immediate	3 days	6.208	0.075
		7 days	6.631	0.051
		14 days	8.091	0.032
	3 days	7 days	0.423	0.733
		14 days	1.883	0.207
	7 days	14 days	1.46	0.232
L4	Immediate	3 days	12.78	0.002
		7 days	12.939	0.027
		14 days	19.032	0.004
	3 days	7 days	0.159	0.95
		14 days	6.252	0.103
	7 days	14 days	6.093	0.01
L5	Immediate	3 days	1.102	1
		7 days	0.788	1
		14 days	5.742	0.075
	3 days	7 days	0.314	1
		14 days	4.64	0.004
	7 days	14 days	4.954	0.001
Edema	Immediate	3 days	5.356	0.007
		7 days	6.98	0.017
		14 days	11.953	0.005
	3 days	7 days	1.624	0.217
		14 days	6.597	0.009
	7 days	14 days	4.973	<0.001

Note. Mean difference = T1 − T2; positive values indicate a reduction in the variable over time. pholm = p-value with Holm correction.

### Healing

Significant changes in healing over time were observed in both groups (p < 0.01), confirming a progressive and sustained improvement throughout follow-up.

However, intergroup comparisons using the Wilcoxon signed-rank test did not reveal statistically significant differences at any of the evaluation points (p > 0.05), as detailed in Table 5. These findings indicate that the progression of healing was predominantly determined by the time factor, without a differential effect attributable to PRF application.

**Table 5:** Temporal evolution of the Landry healing index by intervention group and intergroup comparison at each evaluation point

Time	PRF Group Median (IQR)	Control Group Median (IQR)	p (Wilcoxon)
Day 3	4 [3-5]	3 [3-4]	0.09
Day 7	5 [4-5]	4 [4-5]	0.12
Day 14	5 [5-5]	5 [4-5]	0.3

Note. Data are expressed as median and interquartile range (IQR) according to the Landry healing index (scale 1-5), where 1 = very poor healing and 5 = excellent healing. p = intergroup comparison value using the Wilcoxon signed-rank test.

Despite the lack of statistical significance, the PRF group consistently showed median healing scores equal to or higher than those of the control group at all time points evaluated. In particular, on day 3, the PRF group reached a median of 4 (3-5) compared to 3 (3-4) in the control group, suggesting a possible trend toward an earlier transition to higher-quality healing stages. However, this difference did not remain clinically relevant over time, as both groups converged toward excellent healing scores by day 14.

Taken together, these results suggest that, under conditions of low surgical morbidity, soft tissue healing follows a favorable and predictable course, regardless of the intervention, which could limit the ability to detect additional benefits derived from the use of PRF.

**Figure 3:**
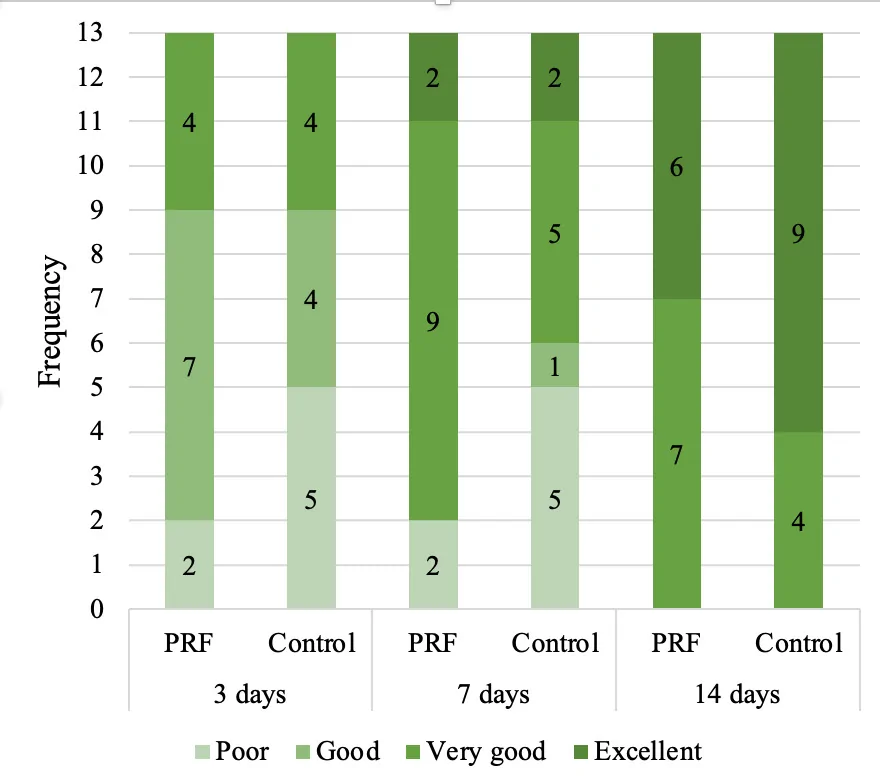
PRF and Control healing (3 days, 7 days, and 14 days).

### Pain

Significant temporal changes in pain were observed in the control group (p < 0.05), whereas no significant temporal effect was observed in the PRF group (p = 0.085).

This pattern may reflect differences in the distribution of pain scores between groups rather than a true differential treatment effect. In the control group, the significant temporal effect (p < 0.05) was consistent with a progressive reduction from higher initial values. In the PRF group, the absence of a significant temporal effect (p = 0.085) should be interpreted with caution, as it may be attributable to the high proportion of zero values reported from the immediate postoperative period onward, which limits the statistical power to detect temporal changes within this group. Although the PRF group showed a trend toward lower pain scores in the early postoperative phase, intergroup comparisons did not reach statistical significance at any evaluation point (p > 0.05), and therefore no differential analgesic effect can be attributed to PRF under the conditions evaluated (Table 6 and Figure 4).

**Table 6:** Temporal evolution of postoperative pain (VAS) by intervention group and intergroup comparison at each assessment point

Time	PRF Group Median (IQR)	Control Group Median (IQR)	p (Wilcoxon)
Immediate	0 [0-2]	3 [2-5]	0.08
Day 3	1 [0-3]	2 [1-4]	0.11
Day 7	0 [0-2]	1 [0-3]	0.15
Day 14	0 [0-1]	0 [0-2]	0.32

Note. Data are expressed as the median and interquartile range (IQR) on the Visual Analog Scale (VAS, 0-10), where 0 = no pain and 10 = maximum imaginable pain. Intergroup comparisons were performed using the Wilcoxon signed-rank test for paired samples. No statistically significant differences were observed between the groups at any evaluation point (p > 0.05).

**Figure 4:**
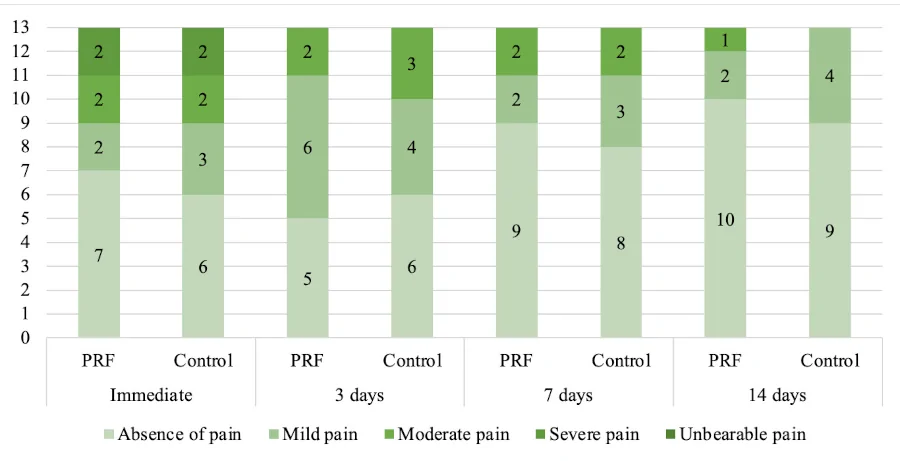
Pain in the PRF and Control Groups (Immediate, 3 days, 7 days, and 14 days).

## DISCUSSION

The results of this randomized clinical trial with a split-mouth design indicate that PRF application in post-extraction premolar sockets does not produce significant differences in inflammation, soft tissue healing, or postoperative pain compared to spontaneous healing. These findings indicate that, under conditions of low surgical morbidity, clinical outcomes are predominantly driven by physiological healing processes rather than adjunctive interventions.

The context-dependent nature of PRF effects is supported by the existing literature. Ozgul et al. ^7^ similarly reported no significant intergroup differences in a split-mouth model, consistent with the present findings. In contrast, Mourão et al. ^16^ and Al-Maawi et al. ^20^ described reductions in postoperative pain in settings involving greater surgical complexity, while Temmerman et al. ^17^ and Srinivas et al. ^18^ highlighted that the clinical benefits of PRF are more evident in procedures with higher surgical trauma or increased regenerative demands. Collectively, this evidence supports the concept that the clinical effectiveness of PRF is strongly dependent on the biological context.

The absence of significant intergroup differences may be explained by a biological ceiling effect. Orthodontic premolar extractions in young and systemically healthy patients typically involve limited surgical trauma, preserved alveolar walls, and inherently favorable healing conditions. Under these circumstances, spontaneous healing may already follow an optimal clinical trajectory, reducing the window of opportunity for biological adjuncts such as PRF to produce detectable additional benefit. Therefore, the lack of superiority of PRF in this model should not be interpreted as evidence of biological inactivity, but rather as an indication that its clinical impact may be limited when baseline healing potential is already high.

From a clinical perspective, these findings do not support the routine use of PRF in low-complexity orthodontic extractions, particularly in young and systemically healthy patients. Its use should be selectively considered in scenarios involving higher surgical morbidity or increased regenerative requirements, where its biological properties may yield clinically relevant benefits.

Future studies should incorporate more sensitive evaluation methods, including histological analysis or three-dimensional imaging, and adopt designs with greater statistical power to more precisely define the clinical indications of PRF across different surgical contexts.

## LIMITATIONS

Despite these findings, several methodological aspects should be considered when interpreting the results. The sample size was relatively small, which may limit the statistical power to detect subtle differences between groups. The study may have been underpowered to detect small intergroup differences. The use of a low-morbidity surgical model in young and healthy patients may have reduced the ability to observe additional clinical benefits of PRF. Clinical outcomes were assessed using macroscopic parameters, which may lack sensitivity to detect early biological changes. In addition, the lack of complete blinding may introduce potential detection bias. The short follow-up period (14 days) restricts the evaluation of long-term healing dynamics. Finally, no three-dimensional imaging or biomolecular markers were included, which could provide deeper insight into tissue regeneration processes.

## CONCLUSIONS

Within the limitations of this split-mouth randomized clinical trial, PRF application did not produce statistically significant differences in soft tissue healing, facial edema, or postoperative pain compared to spontaneous healing in low-complexity orthodontic premolar extractions, a finding consistent with a biological ceiling effect in low-morbidity conditions. Although favorable clinical trends were observed in the PRF group during the early postoperative phase, these did not reach statistical significance. Its safety profile supports further evaluation in higher-morbidity surgical contexts, where its biological properties may yield clinically relevant benefits.
